# Insights into first-principles characterization of the monoclinic VO_2_(B) polymorph *via* DFT + U calculation: electronic, magnetic and optical properties

**DOI:** 10.1039/d2na00247g

**Published:** 2022-08-09

**Authors:** Elaheh Mohebbi, Eleonora Pavoni, Davide Mencarelli, Pierluigi Stipa, Luca Pierantoni, Emiliano Laudadio

**Affiliations:** Department of Materials, Environmental Sciences and Urban Planning, Marche Polytechnic University 60131 Ancona Italy e.mohebbi@staff.univpm.it E.pavoni@staff.univpm.it p.stipa@staff.univpm.it E.Laudadio@staff.univpm.it; Information Engineering Department, Marche Polytechnic University 60131 Ancona Italy l.pierantoni@staff.univpm.it d.mencarelli@staff.univpm.it

## Abstract

We have studied the structural, electronic, magnetic, and optical properties of the VO_2_(B) polymorph using first-principles calculations based on density functional theory (DFT). This polymorph was found to display four optimized structures namely VO_2_(B)_PP_, VO_2_(B)_LP_, VO_2_(B)_PPD_, and VO_2_(B)_LPD_ using the generalized gradient approximation (GGA) PBE exchange-correlation functional by including/excluding van der Waals interaction. Our derivation provides a theoretical justification for adding an on-site Coulomb *U* value in the conventional DFT calculations to allow a direct comparison of the two methods. We predicted a zero bandgap of the VO_2_(B) structure based on GGA/PBE. However, by GGA/PBE + U, we found accurate bandgap values of 0.76, 0.66, and 0.70 eV for VO_2_(B)_PP_, VO_2_(B)_LP_, and VO_2_(B)_PPD_, respectively. The results obtained from DFT + U were accompanied by a structural transition from the metallic to semiconductor property. Here, we verified the non-magnetic characteristic of the monoclinic VO_2_(B) phase with some available experimental and theoretical data. However, the debate on the magnetic property of this polymorph remains unresolved. Imaginary and real parts of the dielectric function, as computed with the GGA/PBE functional and the GGA/PBE + U functional, were also reported. The first absorption peaks of all considered geometries in the imaginary part of the dielectric constants indicated that the VO_2_(B) structure could perfectly absorb infrared light. The computed static dielectric constants with positive values, as derived from the optical properties, confirmed the conductivity of this material. Among the four proposed geometries of VO_2_(B) in this study, the outcomes obtained by VO_2_(B)_PPD_ reveal good results owing to the excellent consistency of its bandgap, magnetic and optical properties with other experimental and theoretical observations. The theoretical framework in our study will provide useful insight for future practical applications of the VO_2_(B) polymorph in electronics and optoelectronics.

## Abstract 2

We have studied the structural, electronic, magnetic, and optical properties of the VO_2_(B) polymorph using first-principles calculations based on density functional theory (DFT).

## Graphical abstract

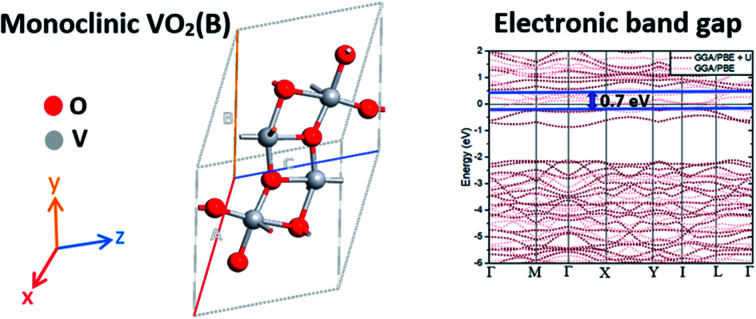

## Introduction

Transition metal oxides^1–3^ have been widely studied during the last decades because of their unique physical properties that are exploitable in the areas of dielectrics,^4,5^ thermoelectricity,^6–8^ catalysis,^9–11^ microelectronics,^12,13^ and thin-film transistors.^14–18^ Vanadium oxides are of particular interest due to these materials provide outstanding advantages in many optoelectronic devices, such as smart windows,^19–21^ sensors,^22–24^ and resistive memories.^25–29^ They have received considerable attention since they are studied as a metal–insulator transition (MIT) material.^30–34^ The MIT can be induced by increasing the temperature/pressure, which causes changes to the structural, electronic, electrical, and optical properties of the materials.^35–42^

According to experimental and theoretical studies, different structures of vanadium oxides have been found at high and low-temperature phases. So far, VO_2_,^43–46^ V_2_O_5_,^47,48^ V_2_O_3_,^49–51^ V_3_O_5_,^52^ V_4_O_7_,^53^ and V_6_O_13_ (ref. 54 and 55) are the most interesting compounds with well known structural properties for the MIT. Vanadium dioxides (VO_2_) are very well known materials with several polymorphs, including tetragonal (R),^56^ monoclinic (M),^57^ triclinic (T),^58^ tetragonal (A),^59^ monoclinic (B),^60^ paramontroseite^61^ and the new body centered-cubic (bcc) structure.^62^ At a high temperature, a metallic phase (VO_2_(R)) with a rutile structure can be achieved, while cooling to below 340 K, the R phase changes into an insulating monoclinic structure M phase.^63–65^ Because the phase transition from the rutile VO_2_(R) and the monoclinic VO_2_(M) is associated with a huge change in resistivity, it has attracted considerable attention for electronic and optical applications.^43,66–69^

VO_2_(B) has been explored as a promising cathode material in Li ion batteries, mainly because of its prominent advantages of high discharge capacity of 323 mA h g^−1^ and low cost.^70–73^ Moreover, the VO_2_(M) and VO_2_(R) phases can be prepared by the irreversible transformation of VO_2_(B) as a precursor.^74,75^

Advanced theoretical and experimental techniques have been implemented to study the VO_2_(B) polymorph. While an experimental bandgap of 0.6–0.7 eV (ref. 76–78) was found for VO_2_ near the semiconductor–metal transition, an X-ray photoelectron spectroscopy study revealed a metallic observation of this material at room temperature.^64^ In agreement with the experimental results, the first-principles calculations confirmed both metallic and insulating features of VO_2_(B).^64,74,79,80^ In the study conducted by Lee *et al.*,^80^ XAS, optical spectroscopy, and DFT calculations were applied to assess the bandgap in the VO_2_(B), VO_2_(M), and VO_2_(A) structures. This study revealed that by comparing the electronic structures of the A, B, and M phases, conventional DFT calculations estimated signatures of a metallic behavior for the A, B, and M phases. Meanwhile, hybrid functional calculations indicated bandgaps of 0.6 eV and 0.5 eV for the M and A phases, respectively, and a very narrow bandgap of 25 meV for the B phase. Zhang *et al.*^74^ investigated the phase transition process from VO_2_(B) to VO_2_(A) based on X-ray absorption spectroscopy (XAS) analysis and DFT calculations. They reported on the metastability of VO_2_(B) in comparison with the VO_2_(A) and VO_2_(R) phases. The calculation of the formation energy in a different phase of VO_2_ showed that the VO_2_(B) structure has less geometrical stability with −6.66 eV formation energy compared to −6.93 and −7.18 eV for VO_2_(R) and VO_2_(M), respectively. This study proposed that the different electronic structure completely depended on the different stabilities of the VO_2_ phases. In a recent study carried out by Popuri *et al.*,^81^ the electron transport properties of the VO_2_(B) structure at low (25–200 K), intermediate (200–320 K), and high temperatures (320–350 K) were investigated using spark plasma sintering. They found different electronic and magnetic properties at different thermal phases. At the low and intermediate temperature phases, nonmagnetic ordering was associated with the insulating characteristic of the structure. At a high temperature, metallic behavior and antiferromagnetic property were observed. In another study, Lourembam *et al.*^82^ used terahertz time-domain spectroscopy (THz-TDS) to investigate the temperature-dependent complex optical conductivity of the VO_2_(B) structure. They observed that VO_2_(B) transformed from an insulating system to a conducting system at 240 K. Furthermore, there was a broad intermediate state with the transition onset being much closer to room temperature, allowing this polymorph to be more suitable for optoelectronic devices near room temperature. In an extensive experimental and theoretical research recently conducted by Wan *et al.*,^83^ they indicated that pure VO_2_(B) has weak absorption in infrared light, with excellent agreement between theory and experiment.

So far, some experimental results have been determined for the geometrical data and MIT for this compound.^82–88^ The electronic properties have been investigated with some experimental and theoretical methodologies; however, the data are still limited and variable.^64,74,79,83,89^ Furthermore, magnetic and optical features are potentially important properties that have not yet been systematically studied for this polymorph with first-principles calculations, and no detailed values have been reported yet. Since a systematic investigation of the efficacy of advanced theoretical methods for computing the chemical and physical properties of VO_2_(B) is missing, our study seeks to fill this gap in the literature. Our present work is focused on the complete theoretical description of the electronic, magnetic, and optical properties of the VO_2_(B) polymorph using GGA/PBE and GA/PBE + U functionals. Recent studies have shown how the combined use of these methods makes it possible to calculate different material properties.^90^ The main objectives of this work are as follows: (i) investigation of the structural parameters of VO_2_(B) by employing different theoretical approaches. The geometry optimization of this polymorph will be assessed by different methodologies in order to find the most accurate results of the material characterization in agreement with the experimental outcomes. (ii) Calculation of the electronic band structure and magnetic properties, (iii) and analysis of the optical properties of the proposed different geometries of VO_2_(B).

## Computational methods

We carried out the atomistic calculations using the Quantum ESPRESSO (QE)^91^ and QuantumATK (QATK)^92^ packages. The DFT approach was implemented in the Kohn–Sham (KS) formulation^93,94^ within the framework of the linear combination of atomic orbitals (LCAO) and plane-wave (PW) basis set approaches, combined with the pseudopotential (PPs) method. PseudoDojo^95^ and Projector Augmented Wave (PAW) PPs^96^ were used for the LCAO and PW calculations, respectively, with the aim to describe the interaction between ion cores and valence electrons. DFT-LCAO and DFT-PW calculations were performed within the GGA framework adopting the PBE exchange-correlation (XC) functional.^97^ Valence orbitals were expanded in a PW basis set with a kinetic energy cut-off of 70 Ry. Brillouin-zone (BZ)^98^ integrations were limited to the gamma point mesh, and a smearing parameter of 0.0001 Ry was considered for the electron population function.^99^ The van der Waals corrections were included by the Grimme's DFT-D3 method,^100^ and the structure was relaxed with the Broyden–Fletcher–Goldfarb–Shanno (BFGS) algorithm.^101^ We implemented both PBE and PBE + U^102,103^ in post-processing calculations in order to make an exhaustive comparison between the different geometries. Since previous studies have shown that the outcomes substantially depended on the magnitude of *U*, we tested different Hubbard *U* values for the vanadium d orbital (*U*^d^) and oxygen p orbital (*U*^p^). In this study, we have set *U*^d^ = 5.20 eV and *U*^p^ = 0.95 eV, wherein they have more similarity to the results reported by Huffman *et al.*^104^ (*U*^d^ = 5.00 eV and *U*^p^ = 0.00 eV). However, our chosen Hubbard values show more agreement between the theoretical and experimental bandgaps, as we will discuss later.

To calculate the electronic band structure and Projected DOS (PDOS), since the KS equation is a nonlinear differential formula, we first converged the charge density with the self-consistent field (SCF) calculations to compute the DOS on a uniform *k*-mesh (we used the 6 × 6 × 6 *k*-points). Then, we ran the non-self-consistent (NSCF) calculation with a twice high *k*-mesh with respect to the SCF calculations (12 × 12 × 12 Monkhorst–Pack mesh) in order to construct the Hamiltonian for the charge density by using the tetrahedron method. PDOS was also considered to account for the magnetic ordering of the compound. Optical calculations were analyzed based on the random phase approximation (RPA).^105,106^ The optical properties of the VO_2_(B) structure in this study are discussed by the two components of the dielectric function (*ε*(*ω*) = *ε*_1_(*ω*) + i*ε*_2_(*ω*)) related to different polarizations in the electric field. The imaginary part of the dielectric coefficient can be obtained from the direct interband transitions through Fermi's golden rule as:^107–109^1
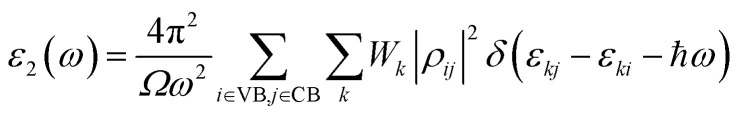
where VB, CB, *ω*, *Ω*, *W*_*k*_, and *ρ*_*ij*_ denote the valence band, conduction band, photon frequency, volume of the unit cell, weight of the *k*-point, and elements of the dipole transition matrix, respectively. Moreover, the real part of the dielectric constant can be obtained using the Kramers–Kronig relation in eqn (2):2
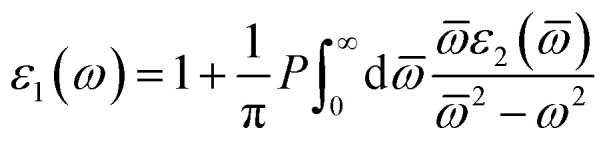
where *P* is the principal value. The real part of the dielectric constants determines the polarization of a material subjected to an external electric field (in this case, the light beam). In addition, the imaginary part shows the amount of light absorption.^110^

The electron energy loss spectrum, *L*(*ω*), can also be described using the dielectric constants by:^111,112^3
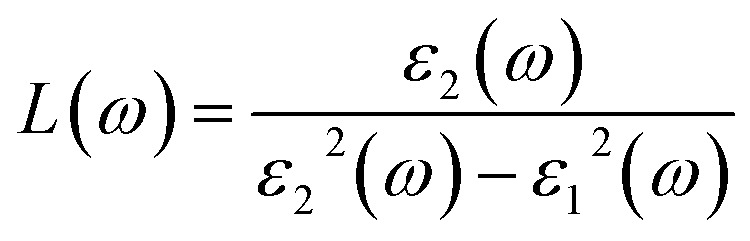


The energy loss function determines the loss of energy while traversing through the material. Molecular graphics were generated using the XCRYSDEN graphical package.^113^

## Results and discussion

### Analysis of the polymeric structure

**Fig. 1:**
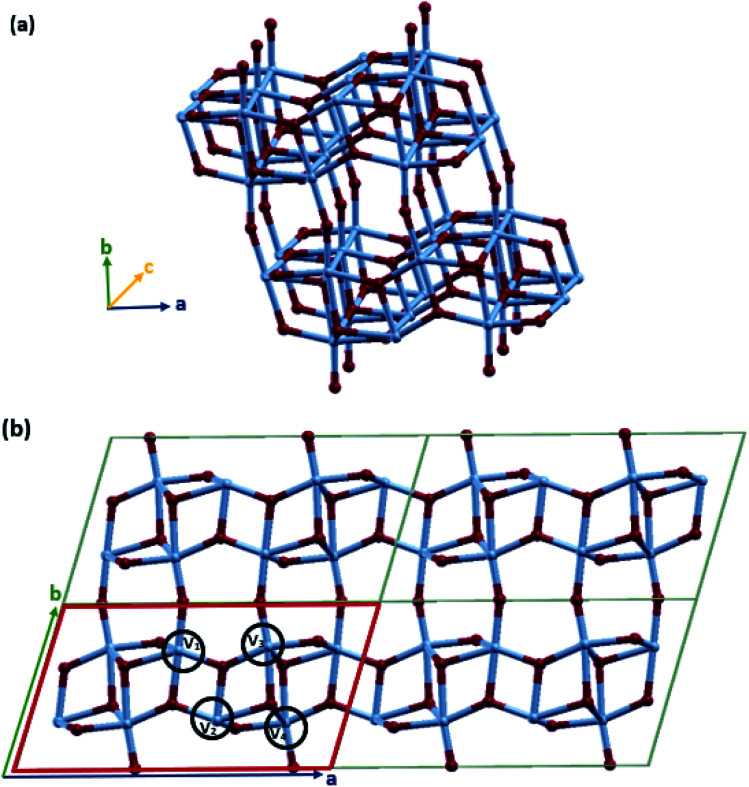
Schematic representations of (a) the 3D frameworks of VO_6_ octahedra in VO_2_(B) (b) 2D 2 × 2 supercell of the VO_2_(B) polymorph, solid red line represents the unit cell of the structure. Color code in the model: V blue and O red.

VO_2_(B) with the space group *C*2/*m* was simulated in periodic boundary conditions (PBC) along with three Cartesian coordinates. As shown in Fig. 1, the base-centered monoclinic unit cell has dimensions of 11.85 × 3.74 × 6.49 Å^3^. This included 12 atoms in the primitive unit cell (4 vanadium and 8 oxygen) and 24 atoms in the conventional unit cell (8 vanadium and 16 oxygen atoms) (see Fig. 1(b)). This unit cell dimension is in good agreement with the previous experimental patterns^64,81,85^ and theoretical studies.^74,79,83^ As shown in Fig. 1(a) and (b), the VO_2_(B) structure can be considered as two identical atom layers including 3D frameworks of VO_6_ octahedra. These octahedra packings of VO_6_ are only linked by oxygen atoms in the corners. The second layer is shifted with respect to the first one by 1/2, 1/2, 0. This polymorph is distorted because of the out-of-center vanadium atoms, resulting in the presence of short/long V–V and two different types of octahedra.

The lattice energy minimized for VO_2_(B) was obtained by employing geometry optimizations of the atomic positions and altering the size and angle of the unit cell, systematically. After the optimization of the lattices, we evaluated the structural parameters and bond lengths on different types of vanadium atoms in the 3D structure. As shown in Fig. 1(b), there are 4 types of V–V bonds in the VO_2_(B) structure. From the experimental bond lengths (see Table 1), they are as follows: (i) the shortest V_1_–V_2_ bond in the center of the *xy* plane with a bond length of 2.89 Å, (ii) V_2_–V_3_ vanadium atoms in the *xy* plane with a length of 3.24 Å, (iii) long V_3_–V_4_ vanadium bonds with an average distance of 3.33 Å, and (iv) medium bond length of V_1_–V_4_ characterized by 3.06 Å.

**Table 1:** Theoretically determined bond lengths of VO_2_(B) optimized structures from the PW and LCAO with and without including the dispersion corrections calculations

Structural parameters (Å)	PW (PBE)	LCAO (PBE)	PW (PBE-D3)	LCAO (PBE-D3)	Experiment^81^
V_1_–V_2_	3.33	2.81	2.98	3.02	2.89
V_2_–V_3_	3.79	3.27	3.34	3.32	3.24
V_3_–V_4_	3.18	3.45	3.50	3.55	3.33
V_1_–V_4_	2.94	3.05	3.18	3.17	3.06

Table 1 indicates that the VO_2_(B) compound exhibits different ranges of the V–V bond distance with the four optimized geometries obtained from the PW and LCAO approaches by including/excluding the dispersion corrections (DFT-D3) in the GGA/PBE calculations. For the convenience of discussion, the experimental and theoretical V–V bond lengths and their differences are listed in Table 1. In comparison to the experimental results in ref. 81, the VO_2_(B) optimized structure obtained from the PW approach and PBE XC functional without including the dispersion corrections (PW(PBE)) (the material named VO_2_(B)_PP_) exhibited V_1_ and V_3_ atoms that were greatly displaced away from the central vanadium atom V_2_ with V_1_–V_2_ = 3.33 Å and V_2_–V_3_ = 3.79 Å. Moreover, V_4_ became closer to V_3_ and V_1_ with distances of 3.18 Å and 2.94 Å, respectively. In the case of PW(PBE-D3) (VO_2_(B)_PPD_) including the van der Waals interactions, the V_1_–V_2_ and V_2_–V_3_ bond distances were shortened to 2.98 Å and 3.34 Å in more agreement with the experimental values of 2.89 Å and 3.24 Å, respectively. However, the V_3_–V_4_ bond distance reached the length of 3.45 Å, which is far from the experimental distance of 3.33 Å. In the VO_2_(B)_LP_ structure obtained from the LCAO(PBE) method, the V_3_–V_4_ and V_1_–V_4_ bond distances were elongated from 3.18 Å and 2.94 Å in VO_2_(B)_PP_ to 3.45 Å and 3.05 Å in VO_2_(B)_LP_, respectively, while the V_2_–V_3_ bond distance decreased from 3.79 Å in VO_2_(B)_PP_ to 3.27 Å in this geometry. In this case, V_1_ and V_2_ significantly became closer together by 2.81 Å, which is less than the experimental value of 2.89 Å. Finally, the optimized structure of VO_2_(B)_LPD_ obtained from LCAO(PBE-D3) including the dispersion corrections exhibited the greatest similarity to the results obtained from VO_2_(B)_PPD_ with distances V_1_–V_2_ = 3.02 Å, V_2_–V_3_ = 3.32 Å, V_3_–V_4_ = 3.55 Å, and V_1_–V_4_ = 3.17 Å.

From the structural parameters computed by the two different PW and LCAO approaches presented in Table 1, we can understand the trend with regards to the treatment of the chosen basis set and XC functional. By using the PW basis set and the PAW XC functional, the approximation tends to overestimate V_1_–V_2_ and underestimate V_3_–V_4_. Conversely, the LCAO basis set associated with the PseudoDojo XC functional leads to an underestimate of V_1_–V_2_ and overestimate of V_3_–V_4_. However, including the van der Waals interactions moderated the system in both approximations. From these results, it is important to note that the basis set approximation can have a significant effect on the computed structures. Calculations on the bulk of similar materials have demonstrated that the LCAO approximation tends to give results that agree less with experimental results compared to the PW. In addition, it is worth noting that van der Waals interactions have a remarkable role in reducing the V_1_–V_2_ and V_2_–V_3_ interlayer distances when we consider our system by the PW basis set (Table 1). Meanwhile, it has the opposite effect when the LCAO basis set is used by enhancing the corresponding bond lengths of V_1_–V_2_ and V_2_–V_3_. As we shall discuss later, since the shortest V_1_–V_2_ bond distance plays a critical role in the electronic structure of the VO_2_(B) polymorphs, the PW(DFT-D3) method gives us more accurate results related to the different physical properties of the VO_2_(B) nanostructure.

In the next step, we calculated the post-processing computations of the electronic, magnetic, and optical properties of four different geometries of the VO_2_(B) polymorph in order to discover which geometry indicates better accordance to experimental and theoretical studies for this material.

### Electronic and magnetic properties

**Fig. 2:**
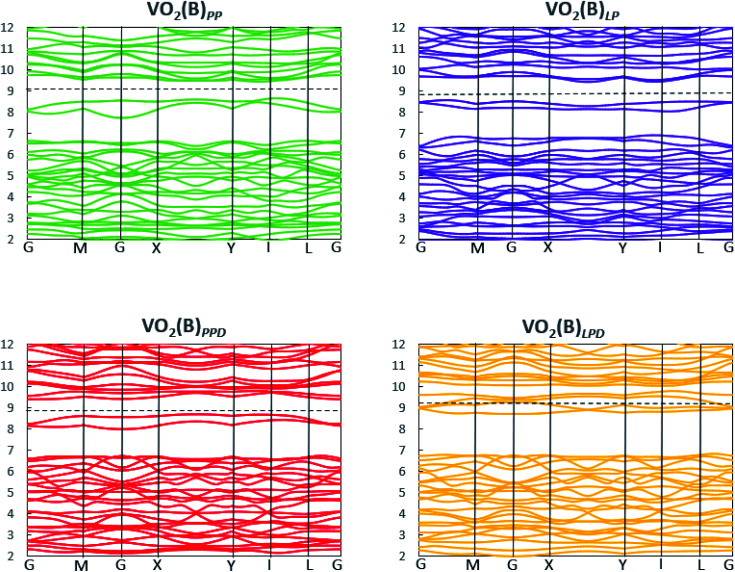
Band structure of VO_2_(B)_PP_, VO_2_(B)_LP_, VO_2_(B)_PPD_, and VO_2_(B)_LPD_ structures predicted by the GGA/PBE + U functional. Fermi energy aligned in the dash line.

**Fig. 3:**
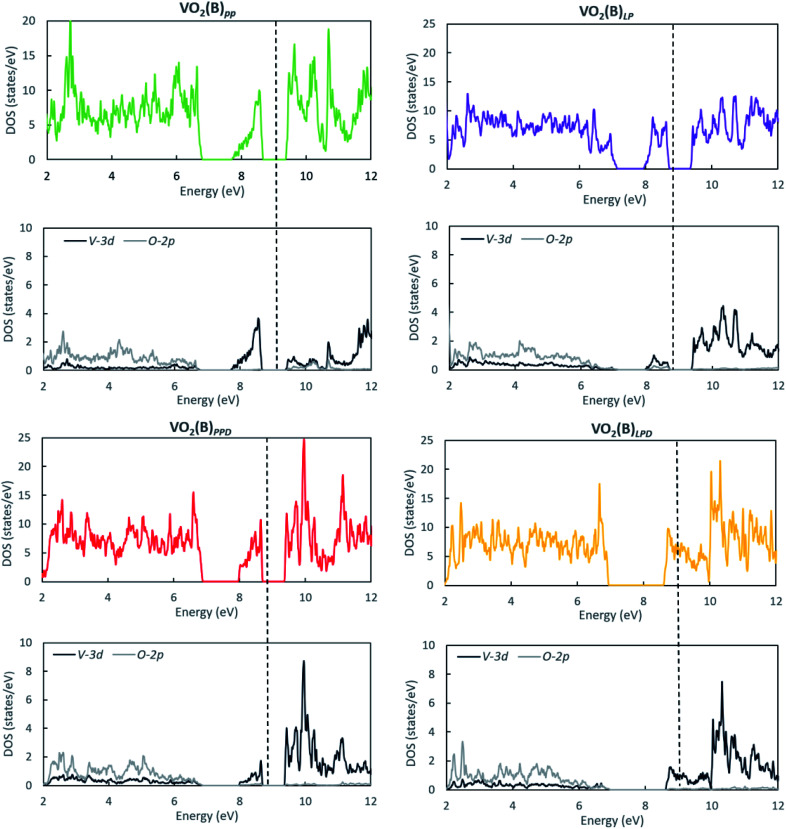
Total and partial DOS curves for the total, V-3d, and O-2p of VO_2_(B)_PP_, VO_2_(B)_LP_, VO_2_(B)_PPD_, and VO_2_(B)_LPD_ structures predicted by the GGA/PBE + U functional. Fermi energy aligned in the dash line.

We studied the electronic properties of the VO_2_(B) polymorph by computing the electronic band structure and the corresponding PDOS curves for total, V-3d, and O-2p for VO_2_(B)_PP_, VO_2_(B)_LP,_ VO_2_(B)_PPD_, and VO_2_(B)_LPD_ geometries based on the PBE and PBE + U approximations. The band structure was analyzed along the high symmetry G–M–G–X–Y–I–L–G directions in the first BZ. The results calculated by GGA/PBE revealed the zero bandgap for all four geometries of the VO_2_(B) polymorph. Since conventional XC functionals such as PBE often underestimate the bandgap in semiconductors,^114^ we also employed the DFT + U method to provide a more accurate prediction of the V d–d orbital correlations and bandgap. So far, the DFT + U method was successful in the prediction of the bandgap for different polymorphs of vanadium oxides. Furthermore, the outcomes agreed relatively well with experimental results.^83,115–118^Fig. 2 and 3 describe the band structure and DOS predicted by the PBE + U functional, respectively. From these results, we found that the Hubbard method described the electronic bandgap in VO_2_(B) well when the U correction effect is considered in DFT. The corresponding bandgap of the VO_2_(B) geometries based on GGA/PBE + U became much larger, *i.e.*, 0.76 eV, 0.66 eV, and 0.70 eV for VO_2_(B)_PP_ (a), VO_2_(B)_LP_ (b), and VO_2_(B)_PPD_ (c), respectively, leading to the semiconductor character of this material. An inspection of Fig. 3 indicates that with the main contribution of total DOS belonged to the V-3d orbital accompanied by less contribution from the O-2p state. As reported in Fig. 2 and 3 (d), excluding/including the U correction in the PBE calculation showed the same zero bandgap for the VO_2_(B)_LPD_ geometry. VO_2_(B)_LPD_ might have a very narrow bandgap. Hence, DFT calculations could not accurately describe the electronic ground states of this structure. Our results from PBE and PBE + U approximations indicate good consistency with the previous experimental and theoretical studies. Our estimated bandgap values by GGA/PBE + U approximation for the optimized structure VO_2_(B)_PP_, VO_2_(B)_LP_ and VO_2_(B)_PPD_ were fitted with the experimental value of 0.6–0.7 eV,^76–78^ comparable with 0.65 eV computed by the HSE method,^74^ and the values predicted by DFT + U (*U*^d^ = 4.00 eV) with 0.60 eV^83^ and 0.78 eV (*U*^d^ = 3.25 eV).^79^ Moreover, the outcomes predicted by Lee *et al.*^80^ revealed the narrow bandgap semiconductor (<25 meV) for the VO_2_(B) structure using the PBE0 hybrid functional.

Next, we examined the magnetism ordering of VO_2_(B) geometries by consideration of the SCF output files and *Lowdin* charge^119,120^ analysis from PDOS calculations.

The magnetism has been reported as two values of the total magnetization and absolute magnetization. While the total magnetization indicates the same value of 4.00 *μ*_B_ for all four geometries of the VO_2_(B) polymorph, the absolute magnetization (Table 2) shows different values of magnetism. As expected, Table 2 reveals that with the Hubbard approximation, the absolute magnetization resulted in higher values than that observed using the conventional DFT, while VO_2_(B)_PP_ and VO_2_(B)_LPD_ showed higher and lower magnetization, respectively.

**Table 2:** Absolute magnetization (in *μ*_B_) of VO_2_(B)_PP_, VO_2_(B)_LP_, VO_2_(B)_PPD_, and VO_2_(B)_LPD_, predicted by GGA/PBE and GGA/PBE + U

Method	VO_2_(B)_PP_	VO_2_(B)_LP_	VO_2_(B)_PPD_	VO_2_(B)_LPD_
GGA/PBE	4.21	4.94	5.10	5.07
GGA/PBE + U	6.01	5.81	5.77	5.55

To elucidate the amount of magnetism contribution of vanadium and oxygen orbitals in the unit cell, we collected the *Lowdin* charges and the magnetic moment (MM) of the V-3d and O-2p orbitals (s and p orbitals for vanadium atoms and s orbitals for oxygen atoms can be neglected because these orbitals have negligible MM contributions). Table 3 indicates the total MM per unit cell (MM/cell) for different geometries of the VO_2_(B) polymorph predicted by GGA/PBE and GGA/PBE + U approximations. With both approaches, the main contribution of the total MM is related to the vanadium 3d orbitals. Inspecting Table 3 reveals that VO_2_(B)_PP_ has increased its total MM by 1 *μ*_B_ for V-3d and 0.77 *μ*_B_ for O-2p orbitals when we included U correction value, while these values have slightly reduced for other geometries by V-3d = 0.47 *μ*_B_ and O-2p = 0.51 *μ*_B_ for VO_2_(B)_LP_, V-3d = 0.38 *μ*_B_ and O-2p = 0.38 *μ*_B_ for VO_2_(B)_PPD_, and V-3d = 0.23 *μ*_B_ and O-2p = 0.15 *μ*_B_ for VO_2_(B)_LPD_. According to the data in Tables 2 and 3, the highest/lowest difference in the total MM/cell between DFT and DFT + U are related to the VO_2_(B)_PP_/VO_2_(B)_LPD_ with the corresponding bandgap of 0.76/0.00 eV. In contrast, the two other VO_2_(B)_LP_ and VO_2_(B)_PPD_ geometries with similar MM have very close bandgaps of 0.66 and 0.70 eV, respectively. The zero bandgap of VO_2_(B)_LPD_ can be attributed to fewer electrons occupying the O-2p orbitals with including the Coulomb repulsive parameter. Therefore, it can be concluded that the metallic state of this geometry is composed of dispersive bands of Vanadium 3d electrons.

**Table 3:** Total MM/cell (in *μ*_B_) for the V-3d and O-2p orbitals of VO_2_(B)_PP_, VO_2_(B)_LP_, VO_2_(B)_PPD_, and VO_2_(B)_LPD_ predicted by GGA/PBE and GGA/PBE + U

Method	VO_2_(B)_PP_	VO_2_(B)_LP_	VO_2_(B)_PPD_	VO_2_(B)_LPD_
V-3d (GGA/PBE)	3.9582	4.3588	4.4502	4.4928
O-2p (GGA/PBE)	0.2904	0.4796	0.5428	1.0296
V-3d (GGA/PBE + U)	4.9598	4.8310	4.8298	4.7266
O-2p (GGA/PBE + U)	1.0654	0.9998	0.9286	1.1864

Since the vanadium 3d orbitals contribute the most to the magnetism of the VO_2_(B) polymorph, we considered the detail of the total MM/cell of the V-3d orbitals. In the transition metal oxides, the d level is fivefold degenerate. The degeneracy of the d level is split into the lower energy t_2g_ level and higher energy e_g_ level by the crystal field splitting in an octahedral field. In this system, the vanadium atom is octahedrally coordinated by oxygen. In the earlier study by Zhang *et al.*,^74^ the semiconducting band structure diagram of VO_2_(B) was precisely explained. It is worth noting that V–V localized pairing interactions influenced the π band and consequently the 3d_*xz*_, 3d_*yz*_ and 3d_*xy*_ orbitals in t_2g_ level. Meanwhile, the 3d_*z*^2^_ and 3d_*x*^2^−*y*^2^_ orbitals (both in the e_g_ level) that are involved in the σ band, are mainly affected by the indirect V–O–V metal–ligand interactions. Table 4 reveals that the electrons predominantly occupy the π band. In contrast, very few electrons occupy the σ band. Based on the outcomes collected in Table 4, it can be observed that the 3d_*xz*_ and 3d_*yz*_ orbitals have the prevailing contribution in the MM/cell, 3d_*xy*_ has some contribution to a lesser extent, while the 3d_*z*^2^_ and 3d_*x*^2^−*y*^2^_ orbitals have negligible contributions. Among the four geometries, VO_2_(B)_LP_ produces quite a different effect. The charge accumulation in the 3d_*xy*_ orbital is more than that for the 3d_*xz*_ and 3d_*yz*_ orbitals (MM = 1.8746 *μ*_B_ for 3d_*xy*_ in comparison to MM = 1.2118 *μ*_B_ and MM = 1.2444 *μ*_B_ for the 3d_*xz*_ and 3d_*yz*_ orbitals, respectively, with PBE + U approximation). In this case, the accumulation of the charge in the 3d_*xy*_ orbitals are greater than those of other geometries. This can be interpreted from the existence of the very short distance V_1_–V_2_ = 1.81 Å (Table 1) in this structure. By comparison, in the other three considered geometries, this bond distance is about 3 Å. Taking into account that the GGA + U method adds a Hubbard-type term to the density functional that increases the electron localization in the correlated orbitals, it is generally believed to provide better results.

**Table 4:** Total MM/cell in *μ*_B_ for the V-3d orbitals of VO_2_(B)_PP_, VO_2_(B)_LP_, VO_2_(B)_PPD_, and VO_2_(B)_LPD_, computed by GGA/PBE and GGA/PBE + U

V-3d	VO_2_(B)_PP_		VO_2_(B)_LP_		VO_2_(B)_PPD_		VO_2_(B)_LPD_	
	PBE	PBE + U	PBE	PBE + U	PBE	PBE + U	PBE	PBE + U
d_*z*^2^_	0.1656	0.1794	0.3682	0.2536	0.2470	0.2280	0.2036	0.2124
d_*xz*_	1.6508	2.0396	1.2102	1.2444	1.6706	2.0778	1.7612	2.1774
d_*yz*_	1.5056	2.2038	1.2514	1.2118	1.5174	1.9674	1.4306	1.8660
d_*x*^2^−*y*^2^_	0.1447	0.2246	0.2146	0.2462	0.2600	0.2776	0.2590	0.2238
d_*xy*_	0.4884	0.3028	1.3146	1.8746	0.7552	0.2792	0.8382	0.2470

According to the experiments carried out by Popuri *et al.*,^81^ macroscopic magnetic measurement results showed that the interactions for the vanadium ions were antiferromagnetic during the high temperature phase. A very weak ferromagnetic property of the VO_2_(B) polymorph can be observed at low temperature. As proposed in this study, the Curie constant (the contribution percentage of the half-spin (*S*_1/2_)) in the vanadium cation is varied in different phases. The obtained curie constant at the low-temperature phase of the VO_2_(B) structure was 12% for *S*_1/2_ in the V-3d cation (spin singlets). This contribution increased to 50% and 100% at the intermediate temperature and high temperature phases (free spins), respectively. Furthermore, experimental X-band EPR spectra in this work revealed a broad resonance line related to the weak interaction of the V–V pairs in the low temperature phase. In contrast, this line became significantly narrower in the intermediate temperature and high temperature phases because of the unlocalized interactions. Similar observations were made by Oka *et al.*,^85^ with the paramagnetic vanadium ions in the high temperature phase and the formation of nonmagnetic V–V pairs in the low temperature phase. In agreement with the outcomes obtained for these studies, our calculations based on GGA/PBE and GGA/PBE + U confirmed the total contribution of 12.5–15.5% for V-3d (as see in Table 3, the MM/cell for V-3d altering between ∼3.95–4.95 *μ*_B_), instead of 32 *μ*_B_ for eight vanadium atoms in the unit cell. These outcomes suggested the presence of less free spins in the VO_2_(B) polymorph, resulting in weak interactions of the vanadium atoms and very poor magnetic (not-magnetic) property of this material. However, the magnetic description of the VO_2_(B) structure has been controversial. Conflicting experimental reports of ferromagnetism,^121,122^ nonmagnetic/antiferromagnetic,^85^ paramagnetic/antiferromagnetic,^81^ and paramagnetic^123^ properties suggest that this material probably has a negligible magnetic susceptibility. We therefore designate it as non-magnetic, as previously reported.^79,81,85,123^

### Optical properties

**Fig. 4:**
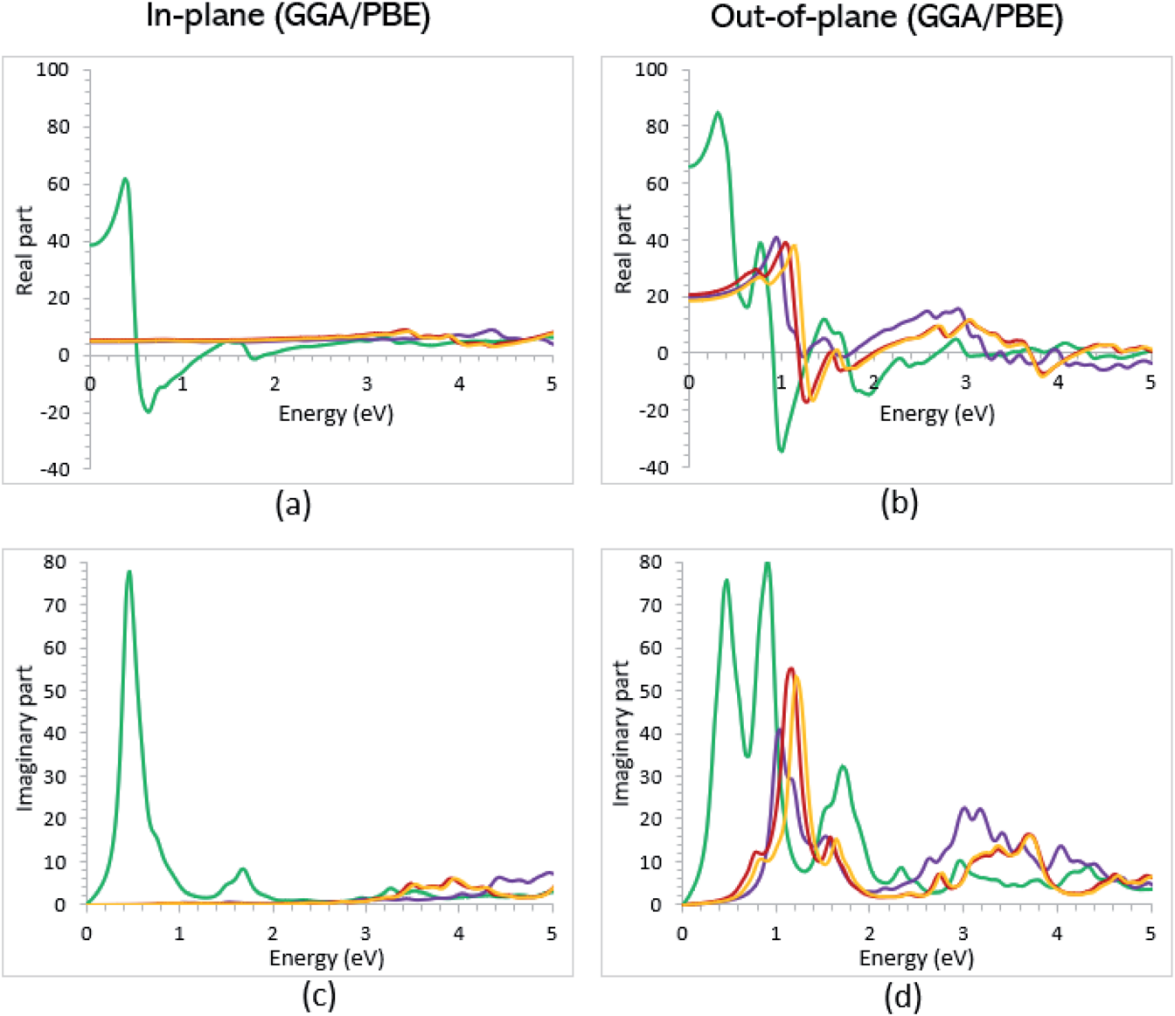
Imaginary and real parts of the dielectric function of VO_2_(B)_PP_ (green), VO_2_(B)_LP_ (purple), VO_2_(B)_PPD_ (red), and VO_2_(B)_LPD_ (orange) structures along the in-plane (a and c) and out-of-plane (b and d) polarizations, as predicted by RPA + PBE.

**Fig. 5:**
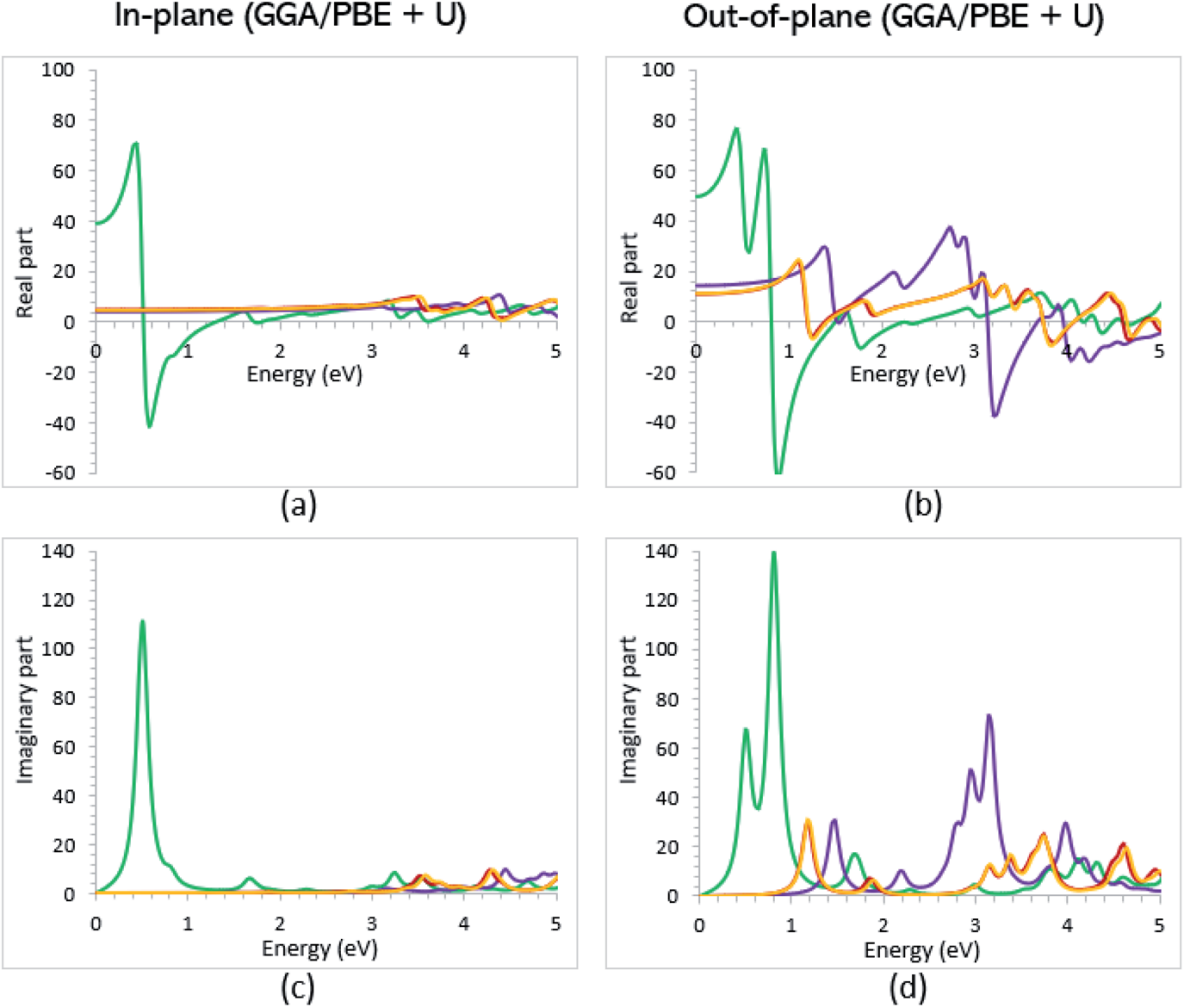
Imaginary and real parts of the dielectric function of VO_2_(B)_PP_ (green), VO_2_(B)_LP_ (purple), VO_2_(B)_PPD_ (red), and VO_2_(B)_LPD_ (orange) structures along the in-plane (a and c) and out-of-plane (b and d) polarizations, as predicted by RPA + PBE + U.

**Fig. 6:**
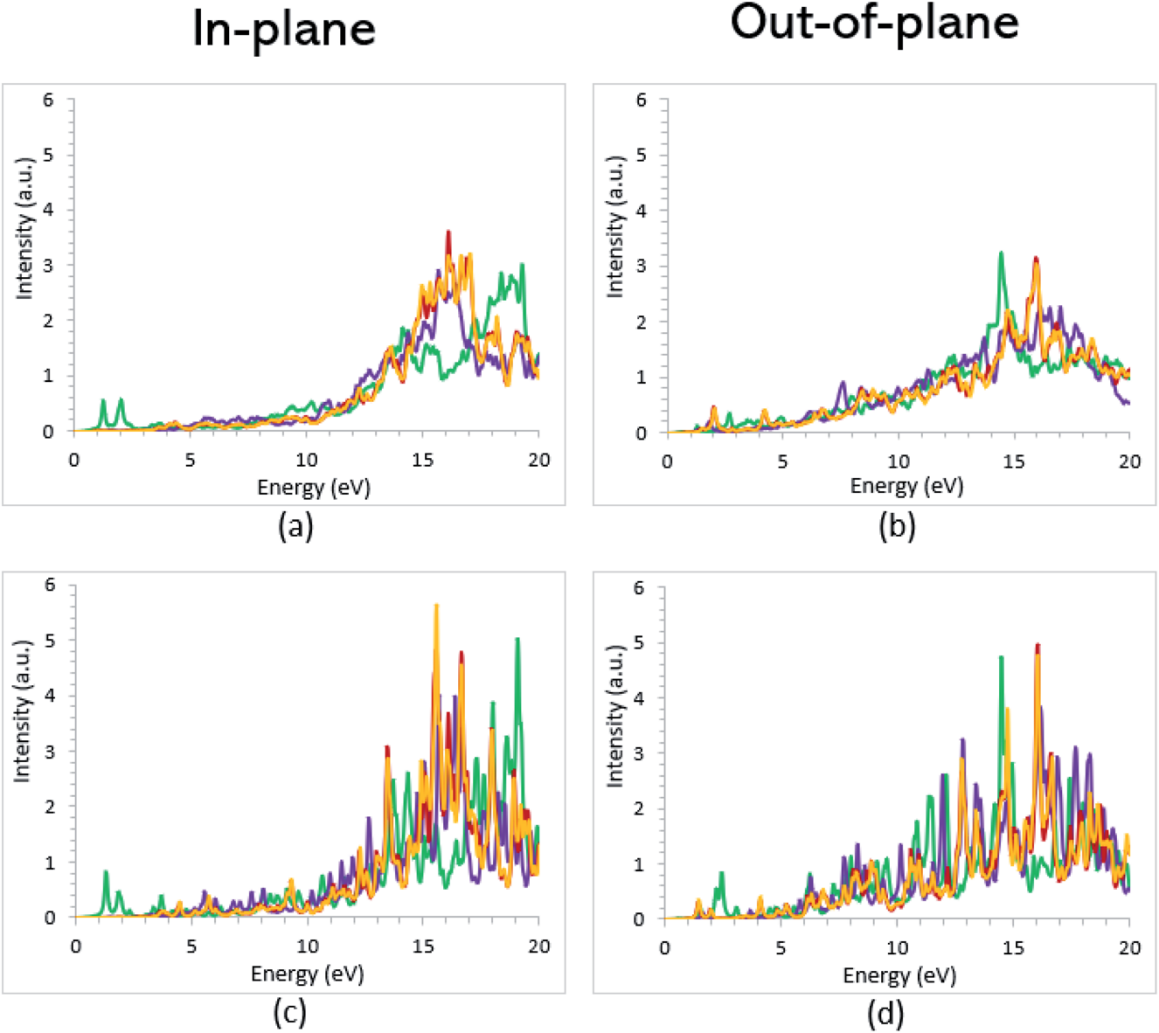
Energy loss function spectra of VO_2_(B)_PP_ (green), VO_2_(B)_LP_ (purple), VO_2_(B)_PPD_ (red), and VO_2_(B)_LPD_ (orange) structures along the in-plane and out-of-plane polarizations, as predicted by RPA + PBE (a and b) and RPA + PBE + U (c and d).

Once the electronic structure calculations confirmed the semiconducting character of the VO_2_(B) polymorph, we probed their optical properties for possible optoelectronics applications. The imaginary (*ε*_2_(*ω*)) and real parts (*ε*_1_(*ω*)) of the dielectric function, as well as the energy loss function for the VO_2_(B)_PP_, VO_2_(B)_LP_, VO_2_(B)_PPD_, and VO_2_(B)_LPD_ structures are presented in Fig. 4–6 as functions of photon energy. We considered the parallel (in-plane) and perpendicular (out-of-plane) polarization directions within RPA + PBE and RPA + PBE + U. According to Fig. 4 and 5, the first main peak of *ε*_2_(*ω*) shows a weak absorption in the infrared range (1.24 meV to 1.7 eV) for the VO_2_(B)_PP_ structure along the in-plane/out-of-plane polarizations. However, the situation changes remarkably for the VO_2_(B)_LP_, VO_2_(B)_PPD_, and VO_2_(B)_LPD_ geometries, in which they indicate that the adsorption peaks in the infrared light are only along the out-of-plane polarizations. Based on the GGA/PBE calculations (Fig. 4), in the case of the in-plane polarization, only VO_2_(B)_PP_ reveals a broad intense peak in the energy range of 0.47 eV, while all considered structures show peaks at 0.46, 1.06, 0.83 and 0.86 for the VO_2_(B)_PP_, VO_2_(B)_LP_, VO_2_(B)_PPD_, and VO_2_(B)_LPD_ geometries, respectively, along the out-of-plane polarization direction. These peaks correspond to the transitions from π → π*. The next highly intense peaks in all geometries are related to the π → σ* transitions. We obtained the optical bandgap of 0.63, 0.56, and 0.60 eV for VO_2_(B)_LP_, VO_2_(B)_PPD_, and VO_2_(B)_LPD_, respectively, along the out-of-plane polarization direction. This is comparable (slightly smaller) to the bulk value of ∼0.6 eV.^77^ For the real part of the dielectric function related to the static dielectric function, it was found that the *ε*_1_(*ω*) part for VO_2_(B)_PP_, VO_2_(B)_LP_, VO_2_(B)_PPD_, and VO_2_(B)_LPD_ geometries shows the positive values of 38.53, 4.60, 5.08 and 5.06 along the in-plane polarization, and 65.99, 19.50, 20.46 and 18.43 for the out-of-plane polarization directions, respectively.

From the predicted data based on the DFT + U calculations, as shown in Fig. 5, the adsorption peaks of *ε*_2_(*ω*) along the in-plane polarization show similar results to the PBE calculations. However, the peaks existing in the optical spectrum of the out-of-plane direction exhibit a blue shift in the light energy range of 1.43 eV for VO_2_(B)_LP_ and an intense peak at 3.10 eV. By applying the U correction in the PBE calculations, the light polarization becomes more intense in VO_2_(B)_PP_, whereas the other three geometries exhibit the opposite behavior by decreasing the peak intensity. Moreover, our theoretical calculations indicate that the optical bandgaps of the VO_2_(B)_PPD_ and VO_2_(B)_LPD_ geometries slightly increase by ∼0.95 eV. Meanwhile, the optical bandgap of VO_2_(B)_LP_ is situated at higher energies at 1.20 eV. A strange behavior is represented by the zero optical bandgap of VO_2_(B)_PP_ at low photon energy. This distinct difference might occur because VO_2_(B)_PP_ contains a longer V_1_–V_2_ = 3.33 Å (more weakly bonded) than the three other configurations with shorter V_1_–V_2_ bond distances of 2.81, 2.98 and 3.02 Å for VO_2_(B)_LP_, VO_2_(B)_PPD_, and VO_2_(B)_LPD_ (Table 1), respectively. The amounts of static dielectric constants were calculated to be 39.32, 4.33, 4.86 and 4.83 along the in-plane direction, and 49.91, 14.55, 10.84 and 10.87 along the out-of-plane polarization direction, with a drop in comparison to the PBE functional. The static optical spectra with the positive value of both in-plane/out-of-plane dielectric constants are further proof of the VO_2_(B) conductivity. Lourembam *et al.*^82^ and Lee *et al.*^80^ experimentally confirmed the non-zero frequency of the real part of the optical conductivity of this polymorph.

As reported in the literature, the different experimental values of the static dielectric constant of VO_2_ have been observed. Yang *et al.*^124^ investigated the temperature dependence of the dielectric constant and carrier conduction in VO_2_ thin films. They outlined that the dielectric constant of VO_2_ can be increased from ∼36 at room temperature to a value exceeding 6 × 10^4^ at 100 °C. In another study, Hood *et al.*^125^ measured the dielectric constant of the VO_2_ structure across the phase transformation at 68 °C. In this work, the real part of the dielectric constant increased from less than 1000 to higher than 90 000 by elongating the film thickness. Furthermore, the outcomes obtained by Mansingh *et al.*^126^ showed the approximated value of 100 for the static dielectric constant of VO_2_ single crystals in the frequency range of 30 to 10^5^ Hz, and in the temperature range 77 to 250 K. From the theoretical side, Wan *et al.*^83^ used both experiment and first-principles PBE + U calculations to investigate the optical property of the VO_2_(B) structure. They observed the weak adsorption of this polymorph in the infrared light along the in-plane/out-of-plane polarization directions. According to the data presented in the literature for the other 2D oxides, VO_2_(B) possesses an excellent dielectric constant along the in-plane and out-of-plane directions. Its dielectric constant is higher than that for Al_2_O_3_ with a value of 8–10 and SiO_2_ with 3.9,^127^ and is comparable with that for HfO_2_ with a dielectric constant of 20–25.^128^ Our calculations indicate that VO_2_(B) can be a good replacement for SiO_2_ with a higher dielectric constant for application in field effect transistors (FETs) and capacitors of dynamic random-access memories. Meanwhile, the stronger infrared absorption of the VO_2_(B) polymorph is favorable for achieving the maximum sensitivity for the applications in uncooled infrared bolometer.^129,130^

The theoretical energy loss function computed by GGA/PBE and GGA/PBE + U is presented in Fig. 6(a)–(d). The energy-loss spectrum is important for describing the energy loss of electrons passing through the materials. While the spectrum calculated by GGA/PBE indicated broad peaks for the in-plane polarization in the energy range of 14–20 eV, GGA/PBE + U indicated in the high intensity peaks along the in-plane and out-of-plane polarization directions. The results reveal that the maximum energy loss peak value predicted by GGA/PBE for VO_2_(B)_PP_, VO_2_(B)_LP_, VO_2_(B)_PPD_ and VO_2_(B)_LPD_ reaches 19.26, 15.99, 16.09 and 16.09 eV along the in-plane polarization direction, and 14.45, 16.96, 15.96 and 15.96 eV for the out-of-plane polarization direction, respectively. The corresponding values predicted by the PBE + U functional are 19.06, 16.32, 16.62 and 15.55 eV along the in-plane polarization direction, and 14.45, 15.92, 16.02 and 16.02 eV along the out-of-plane polarization direction.

According to the theoretical study by Wan *et al.*,^83^ they found an electronic bandgap of 0.60 eV for the VO_2_(B) polymorph. However, the zero optical bandgap was observed in the *ε*_2_(*ω*) optical graph. This disagreement also occurred in our calculations, in which the VO_2_(B)_PP_ structure showed an 0.76 eV electronic bandgap and zero optical bandgap. Conversely, VO_2_(B)_PPD_ indicated a zero bandgap in the band structure calculations and a semiconductor optical property. On the other hand, VO_2_(B)_LP_ was not able to support the correct optical bandgap when the *U* value was included in the PBE calculations. In conclusion, the subtle interplay between the electronic, magnetic, and optical properties leads to the VO_2_(B)_PPD_ configuration describing the semiconductor electronic and optical bandgap well, and shows excellent agreement between experimental and theoretical observations. Therefore, on the basis of the DFT calculations with the PW approach and PBE-D3 method, this configuration strongly suggests a VO_2_(B) polymorph.

## Conclusions

We have successfully reproduced the experimental electronic, magnetic and optical properties of the VO_2_(B) polymorph *via* DFT calculations. In this study, we optimized the geometry of the VO_2_(B) polymorph on the basis of the PW and LCAO approaches using the GGA/PBE functional and with exclusion/inclusion of the dispersion corrections. The analysis of the structural parameters showed the existence of four different geometries of VO_2_(B), namely VO_2_(B)_PP_, VO_2_(B)_LP_, VO_2_(B)_PPD_, and VO_2_(B)_LPD_ obtained from different methods. In order to check for the reliability of the computational methods, particularly for the selected energy functional (PBE + U) with the Coulomb correlation effect, we calculated the electronic and optical bandgaps and magnetic state of the VO_2_(B) configurations for comparison with experiments. The electronic band structure and DOS revealed a zero bandgap for all considered geometries by using the conventional GGA/PBE approximation. However, applying a Hubbard *U* value of 5.20 eV for the V-3d orbitals significantly opened the bandgap up to 0.76, 0.66 eV and 0.70 eV for VO_2_(B)_PP_, VO_2_(B)_LP_ and VO_2_(B)_PPD_, respectively. From these numerical calculations, we indicated that the DFT + U method can be used to change the gap size and induce a metal–semiconductor transition. PDOS solution was used in our potential energy scan and the magnetic properties were assessed. The PBE and PBE + U predicted the nonmagnetic state of the ground-state VO_2_(B) phase, which is consistent with the magnetic moment observed in experiments. Moreover, the optical properties including the imaginary and real parts of the dielectric function for the in-plane and out-of-plane polarizations for the VO_2_(B) geometries were evaluated. The first absorption peaks revealed that all considered geometries can perfectly absorb infrared light along the out-of-plane polarization. Notably, PBE and PBE + U confirmed its VO_2_(B) semiconducting feature with the static dielectric constants having positive values. The DFT-based verification of the nonmagnetic feature as well as the electronic and optical measurements of VO_2_(B)_PPD_, provide the important future research lines to physical characterization of other VO_2_ polymorphs.

## Author contributions

Conceptualization, E. M., E. L. and P. L. S.; methodology, E. M. and E. L.; validation, E. M., E. L. and P. L. S.; investigation, E. M.; data curation, E. M.; writing—original draft preparation, E. M.; writing—review and editing, E. M., E. L., E. P. and P. L. S.; visualization, E. M.; supervision, P. L. S.; project administration, E. M., E. L., P. L. S., L. P. and D. M. All authors have read and agreed to the published version of the manuscript.

## Conflicts of interest

There are no conflicts to declare.

## Supplementary Material
